# Challenges of the set-up process for academic led international studies of rare diseases

**DOI:** 10.1186/1745-6215-16-S2-P196

**Published:** 2015-11-16

**Authors:** Elaine McColl, Kate Bushby, Michela Guglieri, Becky Davis, Gillian Watson, Robert Griggs, Kim Hart

**Affiliations:** 1 Newcastle University, Newcastle upon Tyne, UK; 2 University of Rochester, New York, USA

## 

Researchers and patients agree that rare diseases require new and better therapies. Trials in rare diseases have many challenges, relating to the scarcity of patients and experts, with the inevitable conclusion that large scale studies will require recruitment of patients from multiple centres in different countries. We describe the challenges of setting up an international (US, UK, Canada, Italy, Germany) trial of three steroid regimes in the management of children with Duchenne muscular dystrophy, funded by the US-based NIH(NINDS).

Contract negotiations proved difficult. Differences in the definition of sponsorship between the US and the UK required a major discussion before resolution could be achieved. Risk aversion on behalf of all parties caused major delays. Concerns about exchange rate fluctuations meant that individual researchers were asked to bear the risk of an adverse shift in the exchange. Most of the 40 participating sites requested individual amendments to the model contract, in part because of a lack of compatibility with standard contracts commonly used in the different participating countries; recurrent issues included the currency to be used for payments and who bore responsibility for indemnification.

The discrepancies in the interpretation of the EU Clinical Trials Directive across European countries and the differences between the regulatory and ethical approval processes in Europe, US and Canada meant that we had five separate and distinct timelines and frameworks to deal with in terms of getting regulatory, ethical and local approvals.

A concerted approach is needed to resolve the specific challenges of setting up multicentre studies in rare diseases

